# Intermittent Hypoxic Preconditioning: A Potential New Powerful Strategy for COVID-19 Rehabilitation

**DOI:** 10.3389/fphar.2021.643619

**Published:** 2021-04-30

**Authors:** Ming Cai, Xuan Chen, Jieling Shan, Ruoyu Yang, Qi Guo, Xia Bi, Ping Xu, Xiangrong Shi, Lixi Chu, Liyan Wang

**Affiliations:** ^1^ Shanghai University of Medicine and Health Sciences Affiliated Zhoupu Hospital, Shanghai, China; ^2^ School of Kinesiology, Shanghai University of Sport, Shanghai, China; ^3^ Department of Ultrasound, Huashan Hospital, Fudan University, Shanghai, China; ^4^ College of Rehabilitation Science, Shanghai University of Medicine and Health Sciences, Shanghai, China; ^5^ Department of Pharmacology and Neuroscience, University of North Texas Health Science Center, Fort Worth, TX, United States; ^6^ Shanghai Sunshine Rehabilitation Center, Shanghai, China

**Keywords:** COVID-19, intermittent hypoxic preconditioning, HIF-1α, immune response, inflammatory cytokine storm, rehabilitation

## Abstract

COVID-19 is a highly infectious respiratory virus, which can proliferate by invading the ACE2 receptor of host cells. Clinical studies have found that the virus can cause dyspnea, pneumonia and other cardiopulmonary system damage. In severe cases, it can lead to respiratory failure and even death. Although there are currently no effective drugs or vaccines for the prevention and treatment of COVID-19, the patient’s prognosis recovery can be effectively improved by ameliorating the dysfunction of the respiratory system, cardiovascular systems, and immune function. Intermittent hypoxic preconditioning (IHP) as a new non-drug treatment has been applied in the clinical and rehabilitative practice for treating chronic obstructive pulmonary disease (COPD), diabetes, coronary heart disease, heart failure, hypertension, and other diseases. Many clinical studies have confirmed that IHP can improve the cardiopulmonary function of patients and increase the cardiorespiratory fitness and the tolerance of tissues and organs to ischemia. This article introduces the physiological and biochemical functions of IHP and proposes the potential application plan of IHP for the rehabilitation of patients with COVID-19, so as to provide a better prognosis for patients and speed up the recovery of the disease. The aim of this narrative review is to propose possible causes and pathophysiology of COVID-19 based on the mechanisms of the oxidative stress, inflammation, and immune response, and to provide a new, safe and efficacious strategy for the better rehabilitation from COVID-19.

## Introduction

Since the outbreak and pandemic of the highly infectious and pathogenic COVID-19, most attention has been focused on containing transmission of the coronavirus and addressing the surge of critically ill patients in acute care settings. However, in the future, emphasis will gradually transition to prognosis care and rehabilitation of COVID-19 survivors. Although COVID-19 predominantly affects the respiratory system, it’s anticipated that COVID-19 may have an adverse impact on physical, cognitive, mental and social health status, which is a multisystem disease and frequently severe and often results in death (Barker-Davies et al., 2020; Madjid et al., 2020). Rehabilitation guideline after critical illness recommends progressive rehabilitation programmes are best initiated within the first 30°days (post-acute phase) to have greatest impact on recovery, including improving respiratory function, physical exercise ability, self-care in daily living activities, as well as psychological support, etc (Group, 2009). Very little attention to the prognosis and rehabilitation therapy methods or outcomes of COVID-19 patients after discharge from acute care. So far, only one related study has been published, in which a randomized controlled trail showed that 6°weeks respiratory rehabilitation (2 sessions of 10 min per week) had beneficial to the improvement of respiratory function, endurance, quality of life, and depression following discharge from acute care (K. Liu K.et al., 2020).

The latest epidemiological analysis pointed out a lower incidence of COVID-19 and proposed a possible weaker transmission rate of severe SARS-CoV-2 among high-altitude populations (Arias-Reyes et al., 2020; Segovia-Juarez et al., 2020; Xi et al., 2020). The typical characteristic of plateau environment is hypobaric hypoxia, which could increase tissue oxygen delivery and enhance oxygen utilization. Thus, it can be argued that high-altitude residents may be somewhat tolerant to the consequences of more hypoxemia and systemic tissue hypoxia developing as a result of COVID-19 infection and subsequent lung injury. An intuitive evidence is that hypoxia can reduce the incidence of COVID-19, which may be related to the fact that hypoxia can shorten the half-life of SARS-CoV-2 virus and induce down-regulation of angiotensin-converting enzyme 2 (ACE2) expression, thereby increasing the body’s resistance to viruses (Arias-Reyes et al., 2020). Of note, recently, intermittent hypoxic preconditioning (IHP) as a new non-drug treatment has been used in the clinical treatment of chronic obstructive pulmonary disease (COPD), diabetes, coronary heart disease, hypertension, and other diseases. Many clinical studies have confirmed that IHP can improve the cardiopulmonary function of patients, increase blood oxygen content and the tolerance of tissues and organs to ischemia, inhibit the overactivation of immune system, and control acute pulmonary inflammation. Based on the many beneficial functions of IHP, we speculate that IHP is expected to become a new exploration in the prognosis and rehabilitation of stable COVID-19 cases.

In this review, we briefly outlined the epidemiology, pathological features and histopathology, inflammatory cytokine storm and related damage of COVID-19. Then, we mainly focused on the potential of IHP applied in rehabilitation of COVID-19 and the prognosis based on a variety of IHP related beneficial impacts on physiological functions, and plausible mechanisms to better help the patients to recover and return to society promptly and safely.

## Epidemiology

At December 2019, the first cases of severe acute respiratory infections of unknown origin were reported in Wuhan, China. The causative agent was identified as a novel ß-coronavirus SARS-CoV-2 and the disease was named COVID-19, which is another human infectious disease caused by coronavirus. The transmission of COVID-19 is potent and the infection rate is high. Although the outbreak of COVID-19 is better under control in China, the global situation is still in severe challenge and the confirmed infected cases are continually increased. Since the beginning of 2020, the infection has been spreading worldwide over 215 Countries, causing 115,967,664 cases and over 2,579,775 deaths (as of 09:38 am, March 7, 2021, https://covid19.who.int/), which led the WHO to declare COVID-19 a public health emergency of international concern and the current situation as a new normal for epidemic prevention and control.

The transmission of COVID-19 occurs mainly via respiratory droplets and contact routes, the incubation period ranges from 1 to 14°days with mostly 3–7°days (Guan et al., 2020). All age groups are susceptible, especially the elderly and those with chronic diseases. Now most cases are asymptomatic or mild, but they are potential sources of infection and some patients develop severe pneumonia with acute respiratory distress, septic shock, and multi-organ failure. In other words, asymptomatically infected persons and patients in incubation or recovered from COVID-19 may pose serious challenges for disease prevention and control. The overall case fatality rate is estimated to range from 1 to 16%, which depends on some important parameters such as age, underlying medical comorbidities, preparedness of health system to an outbreak, implementation of preventive measures, and country reaction time to epidemic situation (Verity et al., 2020).

## Pathological Features and Histopathology

The most common symptoms of COVID-19 are fever (98%), dry cough (76%), and myalgia or fatigue (44%) (Huang et al., 2020). Other symptoms are sputum production, arthralgia or sore throat, headache, nausea, vomiting or diarrhea (Borges do Nascimento et al., 2020). Meanwhile, clinical examination shows that severe cases are usually accompanied by obvious hypoxemia (the oxygen saturation is less than 92%) and hypocapnia, which is manifested by decreased arterial partial pressure of oxygen to the ratio of inhaled partial pressure of oxygen and lower plasma CO_2_ levels (Wang D. et al., 2020; Wang M. et al., 2020). Besides, more than half of patients developed dyspnea.

Typical pulmonary imaging findings of COVID-19 cases include multifocal peripherally distributed ground-glass opacities or consolidations, interlobular septal thickening, crazy paving appearance and cystic changes. In autopsies, immunostaining shows bilateral diffuse alveolar damage with cellular fibromyxoid exudates, histological patterns in lung and extrapulmonary tissues were characterized by capillary congestion, necrosis of pneumocytes, hyaline membrane, interstitial edema, pneumocyte hyperplasia, and reactive atypia, which are accompanied by severe inflammatory response (Menter et al., 2020; Xu et al., 2020). Moreover, infiltrates express as macrophages in alveolar lumens and lymphocytes in the interstitium are found in the lung (Song et al., 2020). Again, a lot of findings are suggestive for vascular dysfunction, in lung and other tissues. Meanwhile, COVID-19 has also been shown to be harmful to the heart, some patients are associated with cardiovascular complications such as myocardial injury (Madjid et al., 2020), cardiac arrest (Baldi et al., 2020) and acute heart failure (Tomasoni et al., 2020; Xu et al., 2020). Adverse outcomes of COVID-19 are associated with comorbidities, including hypertension, cardiovascular disease, and lung disease.

Lipids metabolism and inflammation may play key roles in the development of COVID-19. A recent study indicates that lipids are important in the envelopment and transformation of COVID-19 virus, and metabolic disorders may provide additional possibility for the virus to invade host cells (Abu-Farha et al., 2020). The aging associated with increasing chronic inflammation will also destroy the effective control of the immune system in the acute phase of COVID-19 replication (Figliozzi et al., 2020). In addition, COVID-19 will induce excessive inflammation, oxidative stress, abnormal immune responses, and other cardiovascular complications. Then, activation of immune cells will increase oxygen consumption and reduce the supply of O_2_ due to vascular dysfunction, resulting in breathing difficulties and hypoxia, and even death (Cummins et al., 2016).

## Inflammatory Cytokine Storm and Related Damage

Chest computerized tomography (CT) scans of COVID-19 show pneumonia with abnormal findings in all cases, and the potential mechanisms are particularly complex. Clinical and preclinical research will have to explain many aspects that underlie the particular clinical presentations of COVID-19. The data so far are available to indicate that the viral infection is capable to produce an excessive immune reaction in the host. In some cases, a reaction takes place which as a whole is labeled an “inflammatory cytokine storm”, including the tumor necrosis factor a (TNF-α), IL-1β, IL-6, IL-8, IL-12, interferon-gamma inducible protein (IP10), macrophage inflammatory protein 1A (MIP1A), and monocyte chemoattractant protein 1 (MCP1). Moreover, COVID-19 can bind the Toll-like receptor (TLR) to induce the release of pro-IL-1β, which is cleaved into the active mature IL-1β mediating lung inflammation, until fibrosis (Conti et al., 2020). In the early stage of the disease, characteristic laboratory findings of normal white blood cell (WBC) count or mild leukopenia, marked lymphopenia, elevated inflammatory factors (IL-2R, IL-6, TNF-α), suggest that uncontrolled inflammatory responses may further aggravate tissue damage in cardiovascular and other organs (Qin et al., 2020; Wu and McGoogan, 2020). Interestingly, lymphopenia appears to be a negative prognostic factor. The elevated neutrophil-to-lymphocyte ratio (NLR), derived NLR ratio (d-NLR) [neutrophil count divided by the result of WBC count minus neutrophil count], and platelet-to-lymphocyte ratio, can be the expression of the inflammatory storm (Yang et al., 2020). Research has shown that peripheral proinflammatory CD4 and cytotoxic granules CD8 T cells reduce in severe patients, suggesting antiviral immune responses and overactivation of T cells (Xu et al., 2020). However, the reduced T-cell numbers is negatively correlated with IL-6 and TNF-α (Diao et al., 2020). Additionally, the obviously increased levels of senescence markers (PD-1, Tim-3, CTLA-4 and TIGIT) are important signs of severe COVID-19 (Zheng H.-Y. et al., 2020). Moreover, lymphocytes may also become depleted due to the expression of pro-inflammatory cytokines by (not infected) innate immune who are recruited to the lungs and trigger hyper-inflammation, seen during the development of a “cytokine storm” (Cron and Chatham, 2020).

Study shows that excessive inflammation will make patients more prone to endothelial dysfunction and thrombotic diseases in the blood circulation (Bikdeli et al., 2020). Microvascular thrombosis in the pulmonary circulation can lead to an increased dead space. Early pulmonary fibrosis following the disease has been reported from Italy, which could be deficient oxygen-related or excessive inflammation-related. Pulmonary thrombosis has been associated with wedge-shaped infarcts in the lungs on imaging, without the evidence of deep vein thrombosis (Li et al., 2020). Virus also can induce cell death, including necrosis or pyroptosis, proinflammatory cytokine overexpression (uninfected) immune cell recruitment and activation. And COVID-19 may also (partially) escape these mechanisms through the induction of T cell apoptosis (Yi et al., 2020). Pneumonia can lead to respiratory dysfunction and hypoxaemia, which can also bring about cardiomyocyte injury (Zheng Y.-Y. et al., 2020).

## Treatment Strategies

With the rapid increase in the global prevalence and mortality of COVID-19, there is an urgent need to develop targeted therapies. Specific pharmacological treatment for COVID-19 is not currently available. Based on the previously therapeutic experience of SARS and MERS, the potential treatments for COVID-19 include antiviral drugs (anti-HIV drugs, anti-HBV and anti-HCV drugs), plasma transfusion, vaccines and so on (Li and Clercq, 2020). However, now there is no specific antiviral treatment recommended and the effective vaccines are still on the road. Therefore, the clinical efficacy of above drugs requires strictly clinical trials to prove.

In these patients experiencing worsening inflammatory-induced lung injury, there is a decrease in oxygen saturation (<93%). This seems to be the crucial phase of the disease, from this point onwards, there may be a rapid deterioration of respiratory functions. The scenario is truly incredible because, for patients who are paucisymptomatic and slightly hypoxic, the first therapeutic approach is oxygen therapy. A significant number of patients with pneumonia require passive oxygen therapy. Non-invasive ventilation and high-flow nasal oxygen therapy can be applied in mild and moderate non-hypercapnia cases. A lung-saving ventilation strategy must be implemented in acute respiratory distress syndrome and mechanically ventilated patients. Although this strategy is effective, the worsening of respiratory failure may occur in some patients. With the drive preserved, the next step, according to logic, is the non-invasive ventilation (NIV). This therapy has a rapid success by increasing the PaO_2_/FiO_2_ (Partial arterial O_2_ pressure, PaO_2_; Fraction of inspiration O_2_, FiO_2_). In some patients, however, there is a sudden, unexpected worsening of clinical conditions. Patients collapse under the operator’s eyes and require rapid intubation and invasive mechanical ventilation. However, after 24–48 h the patient can have a rapid improvement with an increase in P/F. Operators are therefore tempted to proceed with weaning. But very often, after an initial success, there is a new worsening of respiratory conditions, such as to require a new invasive therapy. Therefore, mechanical ventilation has also been suggested for 1–2 weeks.

The treatment is symptomatic, and oxygen therapy represents the first step for addressing respiratory impairment. Non-invasive (NIV) and invasive mechanical ventilation (IMV) may be necessary in cases of respiratory failure refractory to oxygen therapy. Another clinical trial aiming to test with the use of hyperbaric oxygen is estimated to start on April 25, 2020. The anti-inflammatory effects, which include decreased expression of IL-1β, IL-6 and TNF-α (Aricigil et al., 2018), could be beneficial to mitigate ARDS associated with COVID-19 and fibrosis development.

Although the prospective of counteracting cytokine storm is compelling, a major limitation relies on the limited understanding of the immune signaling pathways triggered by COVID-19 infection. The altered identification of signaling pathways during viral infections may help to unravel the most relevant molecular cascades implicated in biological processes mediating viral infections and to unveil key molecules that may be targeted. Thus, given the key role of the immune system in COVID-19, a deeper understanding of the mechanism behind the immune dysregulation might give us clues for the clinical management of the severe cases and for preventing the transition from mild to severe stages.

COVID-19 has been related to hypoxia and inflammation leading to endothelial dysfunction, increased permeability, and aberrant coagulation in small and large vessels, which are the early hallmarks of organ damage in patients. Moreover, thrombotic complications are a relevant cause of death in COVID-19 patients, and the interaction of SARS-CoV-2 with ACE2 possibly implies alterations of angiotensin II plasma levels. Therefore, the vascular system is increasingly being addressed as a major therapeutic target for defeating COVID-19 (Escher et al., 2020; Giannis et al., 2020).

## Possible Rehabilitation Strategy- Intermittent Hypoxic Preconditioning

In view of the above-mentioned pathological of COVID-19, a non-drug alternative therapy- intermittent hypoxic preconditioning (IHP), via increasing blood oxygen delivery and promoting tissue oxygenation response to improve severe dyspnea, may act as a significant effect on the prognosis and rehabilitation treatment of COVID-19. IHP is a method by which subjects receive exposure to short bouts (1–6 min) of moderate hypoxia (9–12% O_2_), interspersed with the brief periods of normal air (Serebrovskaya, 2002). Firstly, IHP is expected to inhibit the overactivation of immune system, control acute pulmonary inflammation and improve the endogenous reparation for injured tissue, which will become a new exploration in the rehabilitation of stable COVID-19 cases. Secondly, IHP will be an ideal treatment measure of novel coronavirus infection-related acute respiratory distress syndrome. More importantly, IHP has the advantages of high safety, easy-to-accomplish and no side effects.

IHP can trigger the body’s endogenous protective mechanism to make the tissues and cells highly resistant to hypoxia, to relax airways and blood vessels and to improve myocardial contractility. Moreover, it also can increase cardiopulmonary endurance, reduce the area of heart infarction, add blood vessel density and coordinate blood oxygen delivery. Therefore, it can have a strong defense and protective effect on the subsequent longer or more severe ischemia and hypoxia (Neubauer, 2001). Besides, it’s also effective on improving respiratory muscle function and relieving dyspnea, alleviating disease-related anxiety and depression, and enhancing skeletal muscle function of upper and lower limbs (Ponsot et al., 2006; Bao et al., 2020). Studies have confirmed that IHP can improve the balance of the rat’s immune system by activating the defenses of cells against oxidative stress and inflammation (Shi et al., 2015), and IHP is also an effective measure to reduce the damage of the cardiopulmonary system (Ding et al., 2004)

**FIGURE 1:**
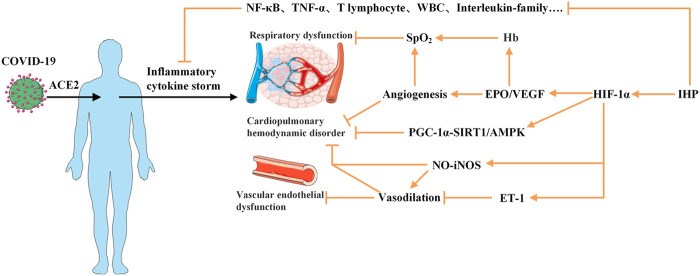
Plausible mechanisms of intermittent hypoxia preconditioning applied for COVID-19 rehabilitation. COVID-19 virus enters the body and combines with ACE2 to produce an excessive immune reaction and to trigger “inflammatory cytokine storm,” which may initiate the pathogenesis of SARS, cardiopulmonary hemodynamic disorder and vascular endothelial dysfunction. Application of IHP on patients may provide inhibitory effect on the levels of various proinflammatory factors and activate HIF-1 to promote target genes to augment EPO/VEGF expression which lead to stimulate production of red blood cell and Hb and angiogenesis to increase capacity to carry oxygen. Furthermore, activated HIF-1 may mobilize PGC-1-SIRT1/AMPK pathway and NO availability and inhibit ET-1. These factors can help reverse the virus induced cardiopulmonary hemodynamic disorder and endothelial dysfunction. ACE2, Angiotensin-converting enzyme 2; AMPK, 5’-AMP activated protein kinase; EPO, erythropoietin; ET, endothelin; Hb, hemoglobin; HIF, hypoxia-inducible factors; PGC, γ-coactivator; NF-B, nuclear factor-k-gene binding; SARS, server acute respiratory syndrome; SIRT, Sirtuin; TNF-α, Tumor Necrosis Factor-α; VEGF, vascular endothelial growth factor; WBC, white blood cell. symbol indicates an activation; symbol indicates an inhibition.

Collectively, combined with the many beneficial functions of IHP, although there is no exact method for the treatment or prevention of COVID-19, we propose that IHP can improve immunity, accelerate the recovery of patients, and reduce the occurrence of positive rejuvenation after discharge. Here, we will explore the potential mechanisms of action of a certain model of IHP in the cardiopulmonary system damage, vascular endothelial dysfunction and hemodynamics of COVID-19, and explored possible rehabilitation options for the prognosis of patients, with a view to providing a novel and effective rehabilitation method for the prognosis of patients, and better help the patients to recover and return to society more promptly and safely (Figure 1).

### Possibility of Applying IHP to the Rehabilitation of COVID-19

Hypoxia is a “double-edged sword”, mainly depending on a variety of the concentration, frequency, and duration of hypoxia exposure, can cause harm or benefit to the human body. Modest hypoxia (9–16% inspired O_2_) and low cycle numbers (3–15 episodes per day) most often lead to beneficial effects, while severe hypoxia (2–8% inspired O_2_) and more episodes per day (48 episodes/day) elicit progressively greater pathology (Navarrete-Opazo and Mitchell, 2014). After the appropriate intermittent hypoxic exposure, tissues and organs can form a complex and active defense mechanism against the same or similar hypoxic environment, and develop resistance and tolerance, thereby to prevent or reduce the damage that it may cause (Semenza, 1999; Verges et al., 2015). This is because that IHP has the function of stimulating the body adaptive physiological changes in different modes of brief repeated exposure in a (10–16% inspired O_2_) hypoxic environment. In the last decade, many clinical trials have described the powerful protective effects of IHP on the respiratory diseases. Rozova et al. confirmed that IHP (15 min 12% inspired O_2_ + 15 min 21% inspired O_2_/cycle, 5 cycle/d for 4 weeks) could relieve the structural damage of the lung air-blood barrier, and promote a specific type of mitosis in lung and heart tissues, then normalize the ultrastructure of lung and heart (Rozova and Mankovska, 2012). Burtscher et al. believe that the protection of IHP on chronic obstructive pulmonary disease (COPD) and coronary heart disease (CAD) benefits from its positive effects of increasing total hemoglobin, enhancing lung diffusion and lung ventilation (Burtscher et al., 2010). Vogtel et al. found that the 18 COPD patients’ exercise endurance enhances, and the functions of forced expiratory volume in 1 s (FEV1), forced vital capacity (FVC) and carbon monoxide diffusing capacity (DLCO) significantly increase, when performing IHP (12–15% inspired O_2_ for 15 times within 3 weeks) (Vogtel and Michels, 2010). Even if IHP is used after brain and spinal cord injury, it can also limit the further development of the disease and promote the remodeling and recovery of tissue structure and function (Baillieul et al., 2017). Many of the previous studies have suggested that IHP has a significant therapeutic effect on COPD and various lung pathological changes. In addition, studies have confirmed that IHP can not only activate hypoxia-inducible factors-1α (HIF-1α) and 5′-AMP activated protein kinase (AMPK)/SIRTl (Sirtuin1)/γ-coactivator 1α (PGC-1α) signaling cascade, but also can reduce the mRNA and protein levels of ACE2, which can significantly inhibit the number of receptors for SARS-CoV-2 virus to enter host cells, thereby to improve endothelial dysfunction, promote cardiovascular hemodynamics, inhibit excessive inflammation and immune response. These changes will be provide the benefit for the recovery from heart and lung injury and dyspnea (Zhang et al., 2009; Singh et al., 2013; Yu et al., 2018 ; Gangwar et al., 2020), and the inhibitory effect of HIF-1α on ACE2 provides a novel idea for using IHP to treat COVID-19.

#### IHP can Enhance the Cardiopulmonary Function

**FIGURE 2:**
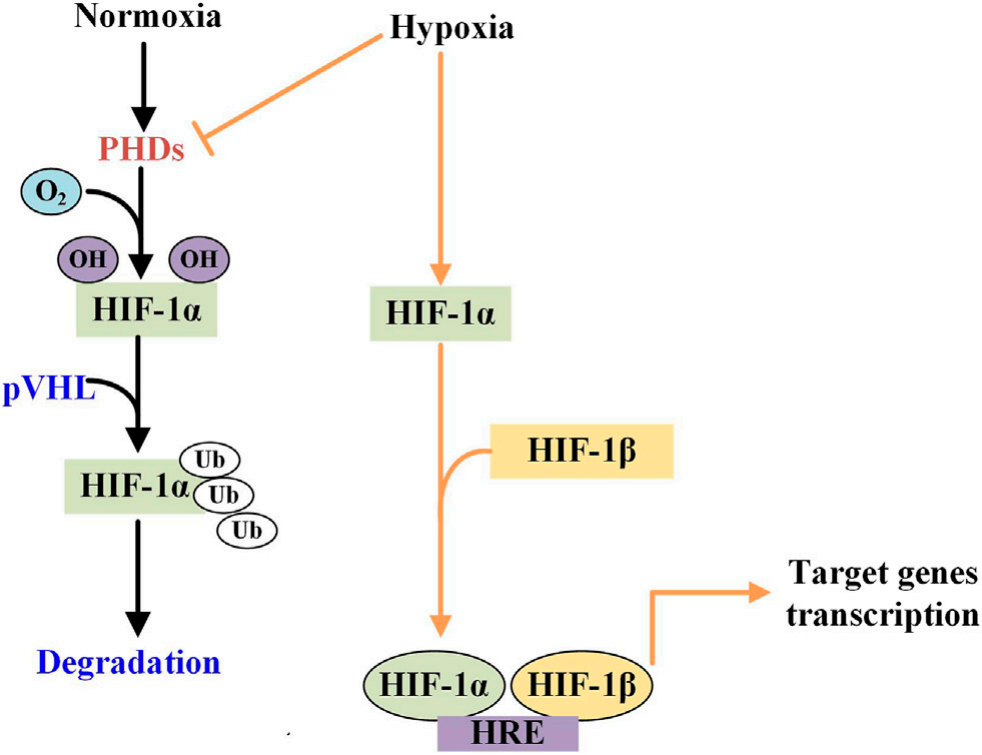
The different effects of normoxia and hypoxiaon the HIF-1α activity. Under normoxic condition, HIF-1 is hydroxylated by prolyl hydorylase (PHD) to attach with von HippelLindau (pVHL) protein and then is degraded. While in hypoxic condition, PHD is inhibited and HIF is phosphorylated which then is translocated and become dimerises with HIF-1β in the nucleus. The heterodimer binds to the hypoxia response element (HRE) to activate the target genes transcription.

It has been showed that ACE2 plays an important role in the immune systems, and SARS-CoV-2 infects host cells through ACE2 receptors causing COVID-19 (Li et al., 2003; Zhou P. et al., 2020). The ACE2 mRNA and protein expression levels of patients with viral infections and complications of cardiopulmonary injury are significantly higher than those of uncomplicated patients, so they have a higher risk of heart disease and critical illness (Chen et al., 2020). Moreover, ACE2 is highly expressed in the heart and kidneys as well as on the lung alveolar epithelial cells, which are the principal target cells for SARS-CoV-2 and the site of dominant injury (Walls et al., 2020). It can be concluded that ACE2-related signaling pathway may play a crucial role in cardiopulmonary injury. It has been fully proven that IHP can reduce and/or reverse cardiovascular damage and cardiopulmonary dysfunction in the way of improving cardiovascular endothelial dysfunction and hemodynamics. This effect usually depends on the HIF-1 mediated downstream signaling pathway. HIF-1 consists of a constitutively expressed subunit ß and an oxygen-regulated subunit α (or its paralogs 2α and 3α). The stability and activity of the α subunit of are regulated by its post-translational modifications such as hydroxylation, ubiquitination, acetylation, and phosphorylation. During normoxia, hydroxylation of two proline residues and acetylation of a lysine residue at the oxygen-dependent degradation domain of HIF-1α trigger its association with pVHL, thereby reducing its stability and leading to HIF-1α degradation (Kaelin and Ratcliffe, 2008). HIF-1 exists in all tissues, with highly sensitive to tissue oxygen tension (Serocki et al., 2018). In hypoxia, the αsubunit becomes stable and will be translocated to the nucleus where HIF-α heterodimerizes with HIF-1β. Then the HIF1α/β complex binds to the promoter regions of target genes containing hypoxia-responsive elements (HRE) and activates a variety of hypoxic adaptive target genes transcription (Figure 2).

IHP-induced hypoxia-reoxygenation cycle can activate HIF-1, thereby promoting the expression of cytoprotective proteins, such as nitric oxide synthase (NOS), erythropoietin (EPO) and vascular endothelial growth factor (VEGF) (Singh et al., 2013), and then up-regulate the synthesis of NO, promote the normalization of microvessels and improve the vasodilation function (Korkushko et al., 2010). At the same time, it can increase the number of red blood cells and improve the vascular microcirculation (Connolly et al., 1989; Krantz, 1991). Some researchers believe that the efficacy of hypoxic adaptation will decrease as patients aging (Levine et al., 1997; Korkushko et al., 2009). However, Korkushko et al. reported that IHP is also well tolerated and safe for elderly patients (Korkushko et al., 2010).

#### IHP can Improve the Vascular Endothelial Function

Endothelial dysfunction is a common feature of cell damage caused by virus infection, which will lead to microvascular dysfunction, in turn, inducing vasospasm or abnormal vasocontraction and thrombosis of multiple major arteries (Steinberg et al., 2012; Chen et al., 2020). Vascular endothelial cells play key roles in regulating vascular tension and peripheral resistance by synthesizing vascular “dilators” (NO etc.) and vasoconstrictors (endothelin-1, ET-1, etc.). The endothelial dysfunction is caused by the unbalanced expression of vascular endothelial factor (Virdis et al., 2011; Lytsy et al., 2013). Gel’tser et al. found that the severity of the disease was positively correlated with vascular endothelial dysfunction in extra-hospital pneumonia, which manifested as insufficient vasodilation or even contraction, and then increased the risk of cardiac hypoperfusion (Gel’tser and Brodskaia, 2005). Aguilar et al. found that applying the treatment of IHP (11.7% inspired O_2_ + 21% inspired O_2_, 4 cycles/d for 4°days) could activate the antioxidant defense mechanisms to the improve the cardiovascular endothelial dysfunction of adult Wistar rats (Aguilar et al., 2018). In addition, study has shown that activation of HIF-1α can inhibit the expression of ET-1 in pulmonary artery smooth muscle cells and reduce pulmonary vasoconstriction (C. C. Wang et al., 2018). Meanwhile, IHP (4–8 h/d, 10%–12% inspired O_2_) can promote the expression of inducible nitric oxide synthase (iNOS) and increase cardiac NO synthesis ability (Coulet et al., 2003; Robinson, et al., 2011). As hypoxia itself can promote the release of NO and other vasodilator factors, it meets the myocardial demand for O_2_ better, which can lead local arterioles to dilate (Park et al., 2015). Lyamina et al. proved that IHP (3 min 10% inspired O_2_+3 min 21% inspired O_2_ for 4–10 cycles) was one of the most effective ways to stimulate the synthesis of endogenous NO, in which the antihypertensive effect was highly correlated to the increase of NO expression level (Lyamina et al., 2011). In addition, Mukharliamov et al. found that IHP (10 cycles/d for 5 min 10–14% inspired O_2_ + 5 min 21% inspired O_2_) combined with antihypertensive drugs significantly reduced the systolic and diastolic blood pressure of hypertensive (Mukharliamov et al., 2006). Furthermore, IHP has been proved not to cause hypertensive reaction in healthy subjects (X. Liu et al., 2017). Faulhaber et al. found that the arterial blood pressure of mild COPD was not be affected during IHP intervention (Faulhaber et al., 2015). Based on these, we hypothesize that IHP can resume the balance between the expression of NO and ET-1 in COVID-19, so as to improve the dysfunction of the cardiopulmonary vascular endothelium and accelerate the recovery.

#### IHP can Improve the Hemodynamics via Activating HIF-1α/EPO/VEGF Signals

The evaluation of hemodynamics is a method to detect whether the blood flow maintains the optimal oxygen delivery, to ensure good oxygen tissue perfusion, and the hemodynamics of COVID-19 may be changed for viral infection. After evaluating the erythrocyte sedimentation rate and high-sensitivity C-reactive protein (hs-CRP) level of 27 COVID-19 cases, Zhou et al. found that the erythrocyte sedimentation rate increased in 18 cases, and the hs-CRP expression added in all 27 cases, which are signs of acute pneumonia or autoimmune system damage (Zhou S. et al., 2020). Some studies also have shown that COVID-19 is characterized by increasing pulmonary capillary pressure, which induces an increase in alveolar-capillary permeability, accompanied by the lung compliance decrease as well as the dead space adding, and even induces cardiopulmonary and other multiple organs dysfunction, ultimately resulting in death.

EPO and VEGF are glycoprotein growth factors regulated by HIF-1, and both play a variety of positive roles in coordinating hemodynamics (Krantz, 1991; Dale et al., 2014). EPO is one of the earliest discovered HIF-1α-regulated target proteins. It is mainly produced in the kidney and used to increase the number of red blood cells. The expression and secretion are closely related to tissue oxygen (Fliser and Haller, 2007). Studies have shown that IHP (6 min 10% inspired O_2_+6 min 21% inspired O_2_, 10 cycles) can activate HIF-1α to up-regulate the EPO activity to provide more O_2_ supply for the myocardia, which in turn stimulates red blood cell production, enhances hematopoietic function and the oxygen-transport capacity (Jelkmann, 1992; Bin-Jaliah et al., 2010). Törpel et al. proved that IHP (3 h, 13.5% inspired O_2_) could increase the central and peripheral EPO levels, and under the same hypoxia intensity, the EPO expression of young people is significantly higher than that of the elders. In addition to promoting red blood cell production, EPO also has other physiological functions (Törpel et al., 2019). Costa et al. treated rats with hypobaric hypoxia and found that the EPO activity was increase induced by HIF-1α. Then, it upregulated the key transcription factor Nrf2 (NF-E2-relatedfactor 2) to exert cellular antioxidant and anti-inflammatory effects, which can promote the synthesis of antioxidant enzymes and reduce the excitotoxicity of nuclear factor kappa-B (NF-κB) induced damage in rat brain and heart (Costa et al., 2013; Mallet et al., 2018). Animal model of myocardial infarction shows that EPO can reduce the infarct size and improve left ventricular function. The mechanism is mainly through the activation of phosphoinositide-3-kinase (PI3K)/protein kinase B (Akt) signal pathways to inhibit cardiomyocyte apoptosis, and mobilize endothelial progenitor cells as well as inhibiting inflammatory cell migration (Roubille et al., 2013).

VEGF, called vascular permeability factor (VPF), the gene expression can be regulated by HIF-1α to increase angiogenesis, improve hemodynamics, and increase the supply of energy substances. Paula et al. found that activating HIF-1α helped to up-regulate the VEGF gene expression for increasing the capillary density (Rodriguez-Miguelez et al., 2015). Senger et al. expounded that intraperitoneal injection of the purified VEGF could increase the vascular permeability of the peritoneal wall, diaphragm, and mesentery *in vitro* (Senger et al., 1983). Connolly et al. found that VEGF could promote the growth of new blood vessels, when injected into the healed rabbit bone graft or rat cornea (Connolly et al., 1989). Dao et al. confirmed that VEGF with exogenous nasal delivery could promote compensatory lung growth in mice, which is manifested by the significant increase of lung capacity and alveolar count (Dao et al., 2018). Fan et al. found that the organism could mediate HIF-1α/VEGF to upregulate the anti-oxidative stress activity during lung injury, which in turn activates the self-protection mechanism of angiogenesis and angioplasty (Fan et al., 2019). In addition, VEGF, cooperated with EPO, can promote angiogenesis, accelerate blood flow, and facilitate the transportation of nutrients and the removal of metabolic waste (Orth et al., 2019). Therefore, we speculate that IHP via the character of activating HIF-1α to upregulate the EPO and VEGF, can reduce the pressure of the vascular circulatory system, increase the efficiency of blood oxygen utilization, and improve the cardiopulmonary circulatory function. As a result, the COVID-19 hemodynamics will be significantly improved and the patient prognostic recovery will be promoted.

#### IHP can Alleviate the Dyspnea via Rectifying Inflammation and Lipid Metabolism Disorders

Evidence suggests that the levels of inflammatory markers, such as IL-6, hs-CRP, immune cells, in the fifth grade level of dyspnea is far higher than the first and second grade despite that the inflammation is not completely related to dyspnea (Garrod et al., 2007; Ryan et al., 2016). In addition, the disorder of lipid metabolism may be the other important factor for dyspnea (Gualdoni et al., 2018). Studies have confirmed that virus infection will interfere with the lipid synthesis and the related signal transduction in host cells. In this process, the virus envelopes and receptors synthesize faster to for completing the virus replication, in consequence, a series of pathological changes occurs in the cardiopulmonary and other peripheral systems (Murillo et al., 2015; Abu-Farha et al., 2020).

In addition to directly activating the HIF-1α signaling cascade, evidences show that IHP can activate the AMPK/SIRT signaling cascade, thereby activating its downstream target PGC1-α, and promoting the dephosphorylation of NF-κB, which is then play a potential protective role in the metabolization and the immune response (Yu et al., 2018; Gangwar et al., 2020). As one of the nicotinamide adenine dinucleotide (NAD+) dependent deacetylases, SIRT1 is a key factor, which is crucial in the regulation of metabolic transcription (H. Yang et al., 2015). Gao et al. confirmed that the increased transcription and expression of SIRT1 in T lymphocytes could better maintain the tolerance of peripheral T lymphocytes, thereby regulating the immune response (Gao et al., 2012). Considering of the close relationship between inflammation, lipid metabolism disorders and dyspnea, IHP may be a potential treatment for improving dyspnea and alleviating tissue hypoxia. For example, in the terms of ameliorating the dyspnea, Berezovskyi et al. performed IHP (1-2w, 3cycles/d, 15 min 12% inspired O_2_+10 min 21% inspired O_2_/cycle) on 55 patients (6–17 years old) with bronchospasm. The results suggest that the bronchial obstruction significantly improves along with the vital capacity enhances and breath-hold time prolongs (Berezovskyi et al., 2015). Serebrovska et al. confirmed that IHP (20 min 12% inspired O_2_+5 min 21% inspired O_2_/cycle, four cycle/session, 5 sessions/w) has little adverse effect on the SpO_2_ of diabetes (Serebrovska T. V. et al., 2019). They also found that the IHP (3 times/d, 15 d, 6–7 min 11% inspired O_2_) significantly increase the alveolar ventilation and maximum lung ventilation of healthy subjects in the sitting and supine positions, and also significantly improve hypoxic ventilation response sensitivity in hyperpnea subjects (Serebrovskaya et al., 1999).

#### IHP can Boost Antioxidant and Antiinflammation Capacity of COVID-19

Gangwar et al. found that the healthy subjects may have a slight inflammatory response in the early stage of IHP (4 h/d, 12% inspired O_2_) implementation. However, the levels of ROS in macrophages tend to decrease, in parallel, the secretion of the antioxidant enzyme enhances, such as glutathione at the 7th-day-IHP treatment, which indicates that the redox homeostasis mechanism is activated to inhibit oxidative stress and acute inflammatory signal response (Gangwar et al., 2020). Rudyk et al. declared that IHP could reduce the accumulation of ROS and inhibit cell apoptosis by enhancing the mitochondria resistance to the open of mitochondrial permeability transition pore (mPTP) in the old rats myocardium heart (Rudyk et al., 2004). Meanwhile, IHP can exert antioxidant protection by inhibiting the excessive formation of reactive metabolites, such as superoxide and peroxide nitrate, or by weakening the activity of stress-activated protein kinase p38 (Ryou et al., 2017). In addition to directly influencing the oxidative stress and inflammatory injury, the IHP likely indirectly reduced these damage via activating the HIF-1α-mediated downstream signals. Tian et al. manifested that the IHP (3 w, 6 h/d 11.1% inspired O_2_ hypoxic exposure) could promote the expression of HIF-1α and the antioxidant ability of kidney to defense the tissue injury in diabetic rats (Tian et al., 2016). This effect may be attributed to the activation of the HIF-1α/VEGF/intranuclear nuclear factor (erythroid-derived 2, Nrf2) signaling pathway, in which the antioxidant enzymes activity enhances to reduce urine protein, inflammatory cell infiltration and glomerular interstitial damage (Chang et al., 2019). Based on the previous studies, we speculate that IHP can improve the inflammation and oxidative stress of COVID-19 by stimulating their own anti-oxidation mechanism.

The effect of hypoxia on the innate immune system and host defenses is controversial. Study has suggested that long-term exposure to hypoxic condition may be harmful to the cellular immune function and increase the risk of respiratory infections at high altitude (Walsh and Oliver, 2016). Wang et al. found that IHP (30 min/d 15% inspired O_2_, 5 d/w, 4 w) could delay the aging of T lymphocyte subsets in the blood, reduce oxidative stress and the production of pro-inflammatory cytokines, and then to the greatest extent to improve immune dysfunction (Wang et al., 2011). IHP can activate the SIRT1 to promote the deacetylation of NF-κB and decrease its activity, thereby reducing the TNF-α and 1L-1β expression to enhance the adaptive immune response (Schug et al., 2010).

## The Possible Application of IHP in the Prognosis and Rehabilitation of COVID-19

### Choose the Appropriate Timing to Apply IHP

All rehabilitation should be carried out under the premise of safety. Patient’s heart rate, arterial oxygen saturation (SaO_2_) and other indicators can be used to determine whether the patient has passed the acute phase. Due to the lack of IHP clinical interventions for COVID-19, the specific cut-in time remains to be further studied. In case a patient shows peripheral capillary oxygen saturation (SpO_2_) < 88% or develops symptoms, such as cardiac arrhythmia, palpitations, sweating, chest tightness, and shortness of breath, which are deemed unsuitable for rehabilitation by the clinician, then the rehabilitation program should be terminated immediately. For mild and moderate cases, rehabilitation interventions should be introduced as early as possible. In contrast, for severe and critical cases, life-saving measures should be prioritized when the patient’s condition is unstable or the disease is still progressing. In such cases, pulmonary rehabilitation interventions should be introduced only when the patient’s condition has stabilized. In addition, in view of safety and human resources, movement of severely or critically ill patients should be limited to their bed or bedside. Once discharged, patients should continue individualized rehabilitation under the premise of strengthening protection and prevention against other infectious diseases such as cold. In addition, the following contraindications should be noted during IHP intervention (Serebrovskaya and Xi, 2016): ① acute phase of physical disease (myocardial infarction within the last three months, unstable angina pectoris, acute ischemic stroke within six months); ② with fever and/or acute infectious diseases and conditions that require enhanced traditional therapy; ③ compensated chronic renal failure, requiring hemodialysis; ④ three-stage hypertension, frequent hypertension; ⑤ severe peripheral blood flow disorder; ⑥ hypercapnic patient; ⑦ congenital abnormalities of the heart and great blood vessels; ⑧ thrombosis and embolism complications; ⑨ primary and secondary polycythemia; ⑩ personal intolerance to hypoxia; ⑪ mental or mental disorders.

### Choose the Appropriate IHP Therapeutic Options

According to the recent research on the cardiovascular protective function of IHP, the use of IHP to improve the prognosis of COVID-19 is traceable. Patients with chronic pulmonary diseases, when exercising, as the interval for red blood cells to pass through the alveolar capillaries is shortened, the ventilation flow rate disorder increases, oxygen intake and blood oxygen saturation decreases. IHP therapy can well make up for the limitations of exercise therapy, not only can ensure the safety and effectiveness of the application, but also be more convenient for home rehabilitation. At the same time, even if the patients with mobility impairments can get the same or even better rehabilitation effect than exercise. Currently, however, there is no standard IHP treatment plan clinically developed. And in recent years, IHP intervention experiments and clinical studies also have large differences in its related parameter settings, such as the range of hypoxic concentration (from 12 to 18%), the duration of hypoxia (ranging from 15 s to 12 h), the number of cycles per day (ranging from 3 cycles to 25 cycles) etc (Serebrovskaya and Xi, 2016). Therefore, it is so important about the strict control of IHP intervention parameters. It also can set parameters according to the hypoxic sensitivity and the development of its disease, such as using the heart rate changes during the steady decline of SaO_2_ to predict the individual’s adaptation to hypoxia and prognosis, and select the best intervention plan.

Based on the above clinical research evidence, we set an IHP plan for the prognosis and rehabilitation of COVID-19: 3–5 min hypoxia (10–16% inspired O_2_) + 5 min normoxia (21% inspired O_2_) as one cycle, 6–10 cycles per day, 3–5 times a week for a total of 8 weeks. Before the formal intervention, the patient should undergo hypoxic preconditioning training to familiarize himself with the treatment equipment and procedures, and at the same time checking the tolerance of the patient and adjusting the appropriate hypoxia concentration. During the treatment process, real-time monitoring of the patient’s heart rate, blood pressure, electrocardiogram, peripheral blood oxygen saturation, lung ventilation function and other physiological indicators. Moreover, as subjects need to inhale low-oxygen gas through a breathing mask, considering the high infectivity of COVID-19, after a patient finished using the device, the disposable face mask should be replaced immediately, and the ventilation pipe and periphery of the device should be disinfected to avoid cross-infection. At the same time, the treatment clinic where the patient is located should be strictly disinfected. In addition, the following principles should be followed: patients should be evaluated comprehensively before starting the rehabilitation program by clinical experts. Evaluation and monitoring should be conducted throughout the IHP rehabilitation program. As COVID-19 patients having chronic pulmonary diseases often have excessive airway secretions, should pay attention to facilitate sputum excretion and reduce the exhaustion due to coughing. Meanwhile, more clinical research should be conducted to prove the safety and efficacy of IHP therapy among COVID-19 rehabilitated phase patients, and explore individualized treatment program.

## Summary and Perspective

Currently, evidence on the prognosis and rehabilitation of COVID-19 patients is insufficient, especially for elderly patients whose disease is complicated by other pathology or comorbidity. It remains unclear whether the impairment of multiple systemic functions is completely reversible or if the long-term existence of the virus can cause residual physical and mental dysfunction in these patients. Nonetheless, we believe that IHP not only has beneficial effect on cardiovascular protection and cardiorespiratory fitness, but also can applied as a potential protector against inflammatory stress. Timely implementing IHP intervention to restore the cardiopulmonary function of COVID-19 and to recover the autoimmunity is very important. IHP intervention program for the prognosis and rehabilitation of COVID-19 should be based on the existing condition and actual vital signs of the patients (such as blood cell parameters, blood oxygen saturation, heart rate, blood pressure, etc.) in consideration of all other underlying disorders (such as hypertension, diabetes, COPD, etc.) to set different intervention parameters to target the biological mechanism mediated by HIF-1α to activate downstream signal targets (iNOS, EPO, VEGF), and at the same time activate AMPK/SIRT1 signaling cascade, and then reduce the tissue and organ damage of patients, and improve the body’s tolerance and resistance to ischemia and hypoxia. It is possible to prevent disease or reduce the virus damage if we may take IHP before attach of virus, which is critical to maintain strong immune capacity, and reduce the prevalence of various chronic diseases. The most appropriate timing and program, the efficacy and safety of IHP for rehabilitation interventions require large-scale clinical trials and further confirmation.
